# Optimized Synthesis of Poly(Lactic Acid) Nanoparticles for the Encapsulation of Flutamide

**DOI:** 10.3390/gels10040274

**Published:** 2024-04-18

**Authors:** Duarte Almeida, Mariana Dias, Beatriz Teixeira, Carolina Frazão, Mónica Almeida, Gil Gonçalves, Miguel Oliveira, Ricardo J. B. Pinto

**Affiliations:** 1 TEMA—Centre for Mechanical Technology and Automation, Department of Mechanical Engineering, University of Aveiro, Campus de Santiago, 3810-193 Aveiro, Portugal; duarte99@ua.pt (D.A.); ggoncalves@ua.pt (G.G.); 2 Intelligent Systems Associate Laboratory (LASI), 4800-058 Guimarães, Portugal; 3 CICECO—Aveiro Institute of Materials, Department of Chemistry, University of Aveiro, Campus de Santiago, 3810-193 Aveiro, Portugal; marianamdias@ua.pt; 4 CESAM—Centre for Environmental and Marine Studies, Department of Biology, University of Aveiro, Campus de Santiago, 3810-193 Aveiro, Portugal; beatrizteixeira99@ua.pt (B.T.); carolina.frazao@ua.pt (C.F.); monica.alm@ua.pt (M.A.)

**Keywords:** biopolymers, poly(lactic acid), nanoparticles, gelification, nanoencapsulation, cytostatic compounds, drug delivery, cancer therapy, nanocomposites

## Abstract

Biopolymeric nanoparticles (NPs) have gained significant attention in several areas as an alternative to synthetic polymeric NPs due to growing environmental and immunological concerns. Among the most promising biopolymers is poly(lactic acid) (PLA), with a reported high degree of biocompatibility and biodegradability. In this work, PLA NPs were synthesized according to a controlled gelation process using a combination of single-emulsion and nanoprecipitation methods. This study evaluated the influence of several experimental parameters for accurate control of the PLA NPs’ size distribution and aggregation. Tip sonication (as the stirring method), a PLA concentration of 10 mg/mL, a PVA concentration of 2.5 mg/mL, and low-molecular-weight PLA (Mw = 5000) were established as the best experimental conditions to obtain monodisperse PLA NPs. After gelification process optimization, flutamide (FLU) was used as a model drug to evaluate the encapsulation capability of the PLA NPs. The results showed an encapsulation efficiency of 44% for this cytostatic compound. Furthermore, preliminary cell viability tests showed that the FLU@PLA NPs allowed cell viabilities above 90% up to a concentration of 20 mg/L. The comprehensive findings showcase that the PLA NPs fabricated using this straightforward gelification method hold promise for encapsulating cytostatic compounds, offering a novel avenue for precise drug delivery in cancer therapy.

## 1. Introduction

Particles at the nanometer scale (nanoparticles, or NPs) exhibit singular features, such as a high surface area-to-volume ratio and customizable optical, electronic, magnetic, and biological properties. Additionally, manipulation of the matter at the nanoscale offers high tunability of their size, shape, chemical composition, surface chemistry, and structure [1,2,3,4], which dictate into what categories they are grouped. Organic NPs have a backbone composed essentially of carbon and generally have a complex structure [2]. Particularly, polymeric NPs are some of the most common due to their high stability in biological fluids and significant efficiency in enclosing drugs, both critical properties in the development of highly efficient drug delivery systems (DDS) [5]. One major advantage of using polymeric NPs is their straightforward surface functionalization, which can increase their stability in circulation and enable their conjugation with active targeting agents [6]. In the past, polymers such as polyacrylamide and polystyrene have been employed to produce polymeric nanocarriers [7]. However, the non-degradability and high toxicity characteristic of these polymers significantly hinders their biological application. Therefore, novel polymers capable of achieving better levels of effectiveness in terms of their biocompatibility and degradation rates have been explored [8]. In this context, the field of nanomedicine has seen an increase in the demand for biopolymers instead of their synthetic counterparts since they are considered more sustainable and renewable alternatives [9]. Additionally, biopolymeric NPs can be degraded by the organism, either via bacteria or metabolic pathways, leading to non-toxic organic byproducts such as CO_2_ or H_2_O [10]. These properties make biopolymeric NPs excellent candidates for use in DDS [11]. Poly(lactic acid) (PLA) is one of the most promising biopolymers that can be produced from sustainable resources, namely corn and sugar cane [11,12]. PLA biodegradation within the human body yields carbon dioxide, water, and lactic acid as degradation products that are safely eliminated through normal metabolic processes [13].

PLA NPs are remarkably versatile and have been used in several fields, from the food industry to biomedicine and tissue engineering [12,14,15], in large part due to their adequate biodegradability and ability to carry active compounds like hormones, vaccines, and anesthetics, which make them particularly popular among other polymeric NPs for controlled drug release [12,16], and even more so for cancer therapy [14,16,17,18]. Simple strategies such as sonication and magnetic stirring have already been proven effective in their preparation, with various experimental conditions having been evaluated [19,20]. However, most articles still emphasize the use of magnetic stirring, which has been shown to consistently lead to a higher degree of particle agglomeration, in comparison to sonication [21,22]. Additionally, the high surface energy of NPs often leads to a common issue in the gelification process, namely the unwanted agglomeration of NPs into micro- or even millimetric sizes. A common strategy to mitigate this issue involves the addition of a second polymer, like PVA, to provide a shell coating that stabilizes the NPs and prevents aggregation [23].

Cytostatic compounds have shown great relevance in cancer chemotherapy research [24]. Unlike cytotoxic compounds, cytostatic drugs do not kill cells but rather act at different cell cycle stages to prevent cell growth [25]. However, their low selectivity leads to numerous undesirable side effects, including immunosuppression and carcinogenesis [26]. Despite the widespread use of PLA NPs, a gap exists in the literature concerning their application as nanocarriers for encapsulating cytostatic compounds like flutamide (FLU), a model compound within this pharmaceutical class that has demonstrated significant efficacy in prostate cancer treatment [27].

In this study, we investigated a straightforward gelification process for synthesizing PVA-PLA NPs via nanoprecipitation to encapsulate FLU, a model cytostatic compound. The size and monodispersity of the NPs were adjusted by evaluating different reaction parameters, including the stirring method, PVA and PLA concentration, and PLA’s molecular weight. The FLU encapsulation efficiency and cell viability were preliminary assessed. By evaluating the effect of parameters like the polymeric molecular weight and stirring methodology—commonly disregarded as essential factors—on the monodispersity, stability, and size controllability of the nanostructure production, this work advances this field and represents a step forward in the development of biobased polymeric nanoparticles capable of efficiently encapsulating cytostatic compounds while controlling the drug pharmacokinetics, thereby reducing significant drug side effects.

## 2. Results and Discussion

**Figure 1:**
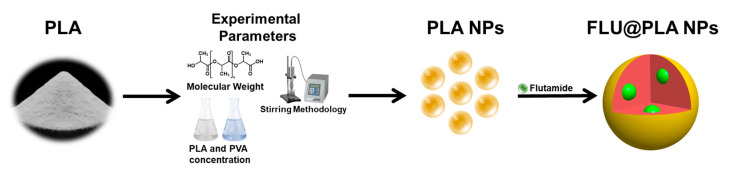
Schematic representation of the synthesis of PLA and FLU@PLA NPs.

In this study, we prepared PLA NPs using a straightforward gelification process. Although the IUPAC definition of NPs simply considers sizes below 100 nm, here, we adopted the widely used terminology for NPs, which is based on the impact of the nanoconfinement on their final properties [28]. The initial evaluation encompassed investigating the impact of several experimental parameters, including the stirring methodology and concentrations of PLA and PVA, as well as PLA’s molecular weight, on the size, polydispersity index, and surface charge of the NPs. Subsequently, under optimized experimental conditions, FLU was efficiently encapsulated within the PLA NPs (Figure 1). Next, we will discuss the influence of several experimental parameters on the controlled gelification of the monodisperse PLA NPs.

### 2.1. Influence of PLA’s Molecular Weight

Variations in the molecular weight of PLA can result in NPs displaying a wide range of properties in terms of their morphologies and sizes [11,14]. For this reason, two PLA polymers with distinct molecular weights, namely PLA HMw (Mw~85,000–160,000) and PLA LMw (Mw = 5000), were explored to investigate their influence on the controlled gelification of the monodisperse NPs.

**Figure 2:**
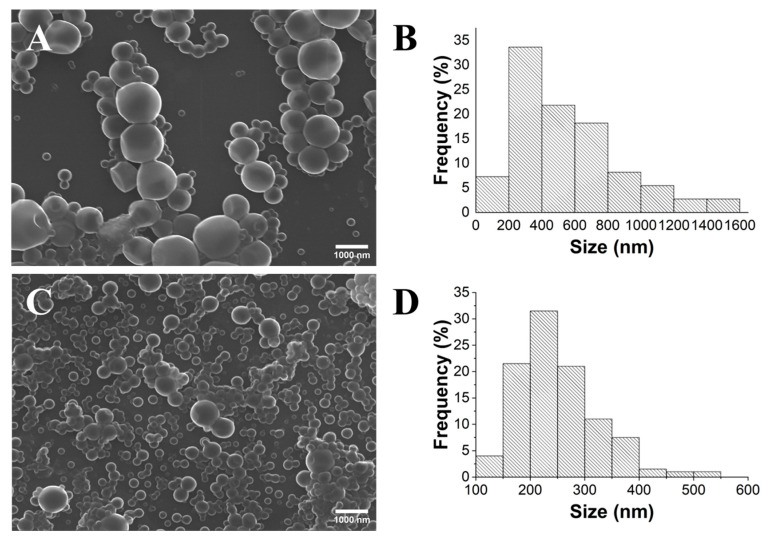
SEM images (×10.0 k) and histograms of the size distribution of the PLA particles obtained using PLA HMw (**A**,**B**) and PLA LMw (**C**,**D**).

The SEM images (Figure 2) clearly demonstrate that PLA HMw yields spherical but highly polydisperse particles, ranging in size from 100 to 1500 nm in diameter. Conversely, it became evident that PLA LMw produces smaller and low-polydispersity particles. It is important to emphasize that PDI values are commonly considered indicative of monodisperse suspensions. According to Filippov et al. [29], the classification of monodisperse particles is considered for PDI values below 0.1. The obtained spherical NPs within the range of 130 to 500 nm presented a mean diameter of 252 nm (±75 nm). This can be attributed to the fact that polymers with a low molecular weight undergo reduced aggregation, leading to increased phase stability [30,31]. Therefore, PLA LMw was chosen for the subsequent evaluation of the other parameters.

### 2.2. Influence of the Stirring Methodology

Initially, three agitation methods were evaluated, namely magnetic stirring (MS), ultra-turrax homogenization (UT), and tip sonication (TS). After the gelification of the NPs, the visual appearance of the suspension clearly indicated that MS was an appropriate approach to generating size-controlled PLA NPs. Numerous aggregates were visible and quickly deposited at the bottom of the sample holder. This phenomenon can be explained by the micrometric size of these gelled aggregates, which may result from inefficient agitation due to a low stirring speed. This fact was already described in prior studies [32], indicating that lower stirring speeds yield large and sometimes aggregated NPs, while higher stirring speeds result in the formation of smaller ones. Contrarily, the samples prepared using the ultra-turrax and tip sonication approaches seemed to generate homogenous solutions. DLS and ζ-potential measurements were conducted to assess the hydrodynamic diameter, polydispersity index, and surface charge of the PLA NPs prepared via ultra-turrax homogenization (PLA UT) and tip sonication (PLA TS) (Table 1).

**Figure 3:**
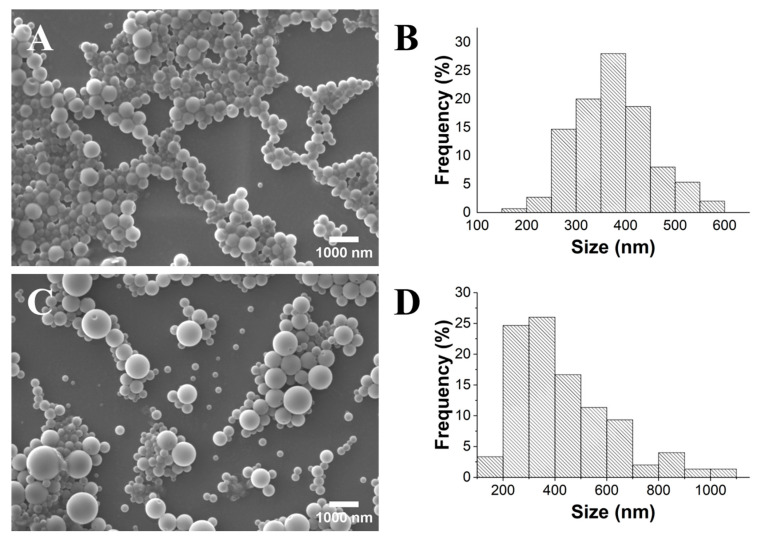
SEM images (×10.0 k) and histograms of the size distribution of PLA UT (**A**,**B**) and PLA TS (**C**,**D**) samples.

The hydrodynamic size values obtained for the samples PLA TS and UT were 538 and 365 nm, respectively. The NPs synthesized using the UT method exhibited the smallest size and a PDI of 0.141, which can be attributed to the higher stirring speed achieved by using this gelification process. Indeed, some authors argue that a threshold of 0.3 is more suitable for polymeric nanosystems intended for pharmaceutical applications [33]. However, a remarkable difference was observed concerning the surface charge of the PLA NPs obtained using these two methods. The PLA UT and PLA TS samples showed ζ-potential values of −10 mV and −26 mV, respectively. It is widely recognized that the absolute value of the ζ-potential directly correlates with the stability of a suspension, with values below −30 mV and above +30 mV typically indicating very stable suspensions [34]. Although the values in both cases were higher than −30 mV, the PLA TS NPs exhibited a notably higher stability. To further validate the size and morphology of the fabricated NPs, a detailed SEM analysis was carried out (Figure 3).

The PLA UT NPs displayed a size range of 180 to 590 nm, with a mean diameter of 374 nm (±77 nm), whereas the PLA TS NPs exhibited a size range of 160 to 1050 nm, with a mean diameter of 434 nm (±189 nm). In both cases, the samples showed a spherical morphology, with their size and respective standard deviation comparable to those determined in the DLS analysis (Table 1). However, the PLA TS sample appeared to have more dispersed particles, which may justify the ζ-potential value obtained. The SEM micrograph of PLA MS (Figure S1) reveals numerous clustered particles of micrometric sizes without a defined morphology, confirming the results observed through visual analysis. Thus, the UT and TS methods were shown to be more effective than the MS approach for the gelification of lower-polydispersity PLA NPs. The UT method enables the gelification of a slightly narrower and less polydisperse distribution of NPs compared to the TS method. However, the significantly more negative ζ-potential value associated with the TS method makes it the ideal approach for preparing long-term stable NPs. This fact is in accordance with previous works, in which sonication led to smaller and more less polydisperse distributions of particles [21,22]. This effect is attributed to cavitation, in which collapsing microbubbles disrupt the formation of covalent bonds in polymers [35]. Comparatively, Sumitomo et al. [36] observed a more efficient particle dispersion with ultrasonic irradiation than mechanical stirring at a lower input power. However, this dynamic reversed at a higher input power (after an increase in ultrasonic frequency and number of rotations per minute).

### 2.3. Influence of PLA Concentration

**Figure 4:**
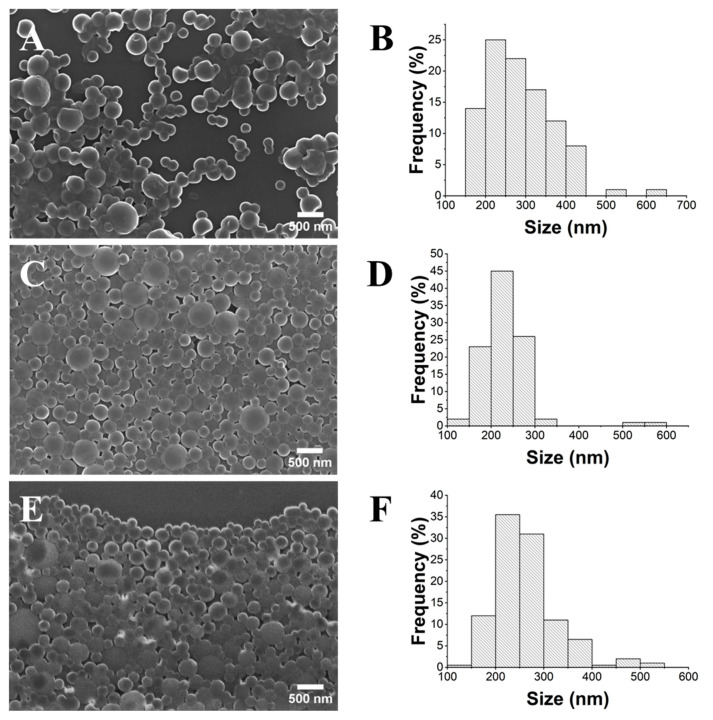
SEM images (×20.0 k) and histograms of the size distribution of PLA_1_ (**A**,**B**), PLA_10_ (**C**,**D**), and PLA_50_ (**E**,**F**).

For the optimization of the PLA NP gelification process, three different initial concentrations were explored: 1, 10, and 50 mg/mL (PLA_1_, PLA_10_, and PLA_50_). For PLA_1_, SEM analysis (Figure 4) showed a size distribution in the 165–650 nm range, with an average diameter of 290 nm (±85 nm). For the samples PLA_10_ and PLA_50_, NP diameter ranges of 175–300 nm and 200–330 nm were obtained, respectively. Importantly, the PLA_10_ sample was the only one that fulfilled the criterion of monodisperse NPs (PDI < 0.1) [29].

As seen in Table 2, the DLS tests confirmed this correlation between PLA content and size distribution, despite showing higher values for the average diameter and much narrower ranges. However, it was reported that the ζ-potential of the prepared PLA NPs increased in its absolute value from the lower-concentration solutions to the higher-concentration solutions of PLA: −18.4 mV (PLA_1_), −13.4 mV (PLA_10_), and −12.6 mV (PLA_50_).

The characteristic negative ζ-potential of the PLA NPs can be mainly attributed to the oriented carboxylic acid groups on their surface due to polar interactions with the aqueous medium. According to Negi et al. [37], a negatively charged value for PLA NPs is mainly governed by the establishment of an equilibrium between the acid and the carboxylate, which is pH-dependent. Therefore, the final ζ-potential value of the PLA NPs is highly dependent on several intrinsic properties, such as their size distribution, aggregation state, and composition [19]. Therefore, smaller and monodisperse PLA NPs were expected to present a larger surface area and a higher exposure to carboxylic acid, consequently resulting in a more negative ζ-potential value. The PLA concentration increase from 1 to 10 mg/mL marks a decrease in the pH from 6.50 to 6.17, likely as a result of an increased concentration of carboxylate groups. Despite this, the absolute value of the ζ-potential decreased. This can be attributed to the protonation of the exposed carboxylic acid groups, which were fewer in quantity due to the PLA-PVA interface formed as a result of the carboxyl–hydroxyl hydrogen bonds [37]. A PLA content of 50 mg/mL, however, did not see a significant impact on the pH or ζ-potential but resulted in a slight increase in the average diameter and a significant increase in the PDI value. This suggests that around this concentration, the amount of PLA in the solution might exceed the coating capacity of the PVA, diminishing its stabilizing effect due to the increase in the exposed carboxylic groups from the PLA [38]. Due to the smaller size distribution of the PLA NPs obtained for the sample with an initial concentration of 10 mg/mL, as confirmed by SEM and DLS analysis, this condition was deemed the most suitable for the controlled gelification of monodisperse PLA NPs.

### 2.4. Influence of the PVA Concentration

**Figure 5:**
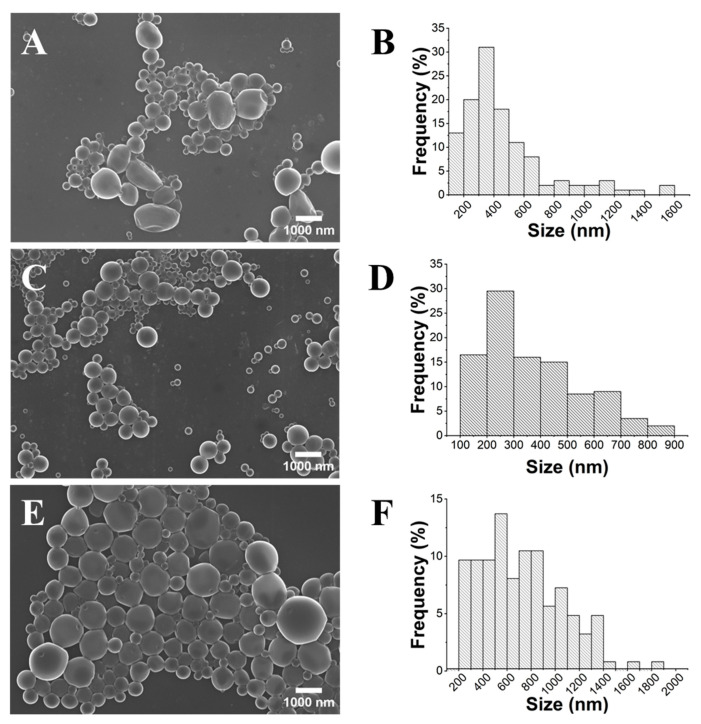
SEM images (×10.0 k) and histograms of the size distribution of PVA_0.1_ (**A**,**B**), PVA_2.5_ (**C**,**D**), and PVA_10_ (**E**,**F**).

The influence of the surfactant concentration on the gelification process of the PLA NPs was also investigated. In the absence of PVA, a dense gel is formed (Figure S2). This effect can be attributed to the high surface tension exerted by the different phases because of intermolecular interactions [39], highlighting the importance of the use of surfactants in the preparation and stabilization of PLA NPs. Therefore, three different concentrations of PVA (0.1, 2.5, and 10 mg/mL) were studied. It was noticed that all the PVA-including samples (PVA_0.1_, PVA_2.5_, and PVA_10_) formed homogeneous suspensions. Despite having formed a homogeneous suspension, the sample PVA_0.1_ showed in the close SEM analysis the presence of remarkably large agglomerates (Figure S3A). In contrast, the SEM analysis of the suspensions formed in PV_2.5_ and PVA_10_ (Figure 5) showed that the former had much smaller NPs and a narrower size distribution (110–850 nm) than the latter (210–1900 nm).

The increased particle diameter seen in PVA_10_ was likely the result of a thick PVA coating. As a surfactant, there is a critical concentration of PVA above which further adsorption only results in a diameter increase without a noticeable effect on the particle stability [38]. According to DLS (Table 3), higher PVA contents also appear to have resulted in a lower ζ-potential between PVA_0.1_ and PVA_2.5_ but with little effect after further addition of the surfactant.

As the PVA content increased, the ζ-potential was shown to visibly decrease in value, despite the pH remaining relatively constant. As such, this decrease appeared to be directly dependent on the composition. The correlation between PVA and the ζ-potential has been studied before and is attributed to the presence of acetate groups [40]. Kleimann et al. [41] observed a positive correlation between the adsorption of PVA onto poly(styrene sulfate) latex particles and charge neutralization. This coincides with the present results since the magnitude of the ζ-potential decreased when the PVA content rose from 0.1 to 2.5 mg/mL. Only above 2.5 mg/mL was the diameter seen to increase visibly, similar to what the SEM images in Figure 5 show. Following this analysis, 2.5 mg/mL was considered the optimal concentration of PVA moving forward, as it allowed the lowest values for the mean diameter and PDI.

### 2.5. Synthesis of the FLU@PLA NPs and Determination of the Encapsulation Efficiency

**Figure 6:**
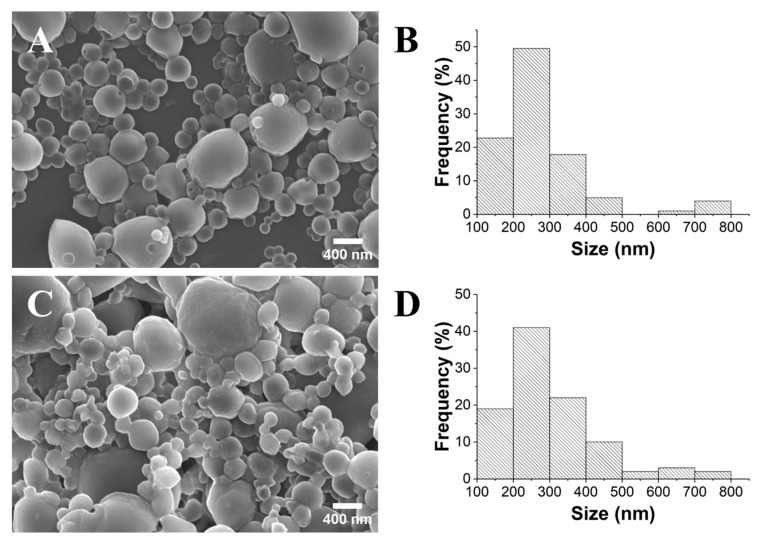
SEM images (×25.0 k) and size histograms of pristine PLA NPs (**A**,**B**) and FLU@PLA NPs (**C**,**D**).

The encapsulation of FLU into the PLA NPs was undertaken using the optimized gelification parameters alongside the addition of PEG. Coating the surface of NPs, such as PLA NPs, with PEG is widely used for encapsulating compounds, providing enhanced stability, biocompatibility, controlled release, and surface modification, making it a common strategy in drug delivery and biomedicine [42]. Furthermore, it can prevent interactions between the particles, reducing the degree of aggregation [43]. The resulting suspension exhibited a yellow tone, characteristic of FLU, giving a first indication of the successful encapsulation of this cytostatic compound. Based on the SEM images and the size distribution histograms presented in Figure 6, it appears that there were no significant differences between the pure PLA and FLU@PLA NPs. Both samples exhibited NP diameters mainly within the range of 100 to 500 nm (>90%). However, it was verified that there was a slight increase in the mean diameter from 276 nm (±132 nm) to 306 nm (±152 nm) after the FLU encapsulation. This size enhancement was another form of validation of FLU’s presence and encapsulation effect, also verified by other authors for α-tocopherol [44] (and docetaxel-loaded PLA-modified NPs [45]). Interestingly, the impact of the PEG coating on the NPs is evident from the SEM images, confirming that while a spherical morphology is preserved, it becomes more irregular. Additionally, the surface of the NPs appears less smooth, displaying a more granular structure (Figure S4).

The amount of encapsulated FLU was then determined using UV-Vis spectroscopy since FLU absorbs radiation in this region of the light spectrum. The peak at 300 nm was chosen as it was not pH-dependent [46]. Consequently, a calibration curve (Figure S5) was constructed to enable the calculation of the percentage of FLU encapsulated. The concentration was obtained based on the concentration of FLU “free” in each washing step (Table S1). Approximately half of the FLU (50.8%) was retrieved in the initial wash. This could be attributed to the high amount of FLU used in this assay (25 mg). However, in subsequent washing steps, the remaining FLU in the solution seemed to be minimal. Overall, 56.1% of the FLU was found in the supernatants, indicating an encapsulation degree of 43.9% in the PLA NPs.

### 2.6. Cell Viability Assay

**Figure 7:**
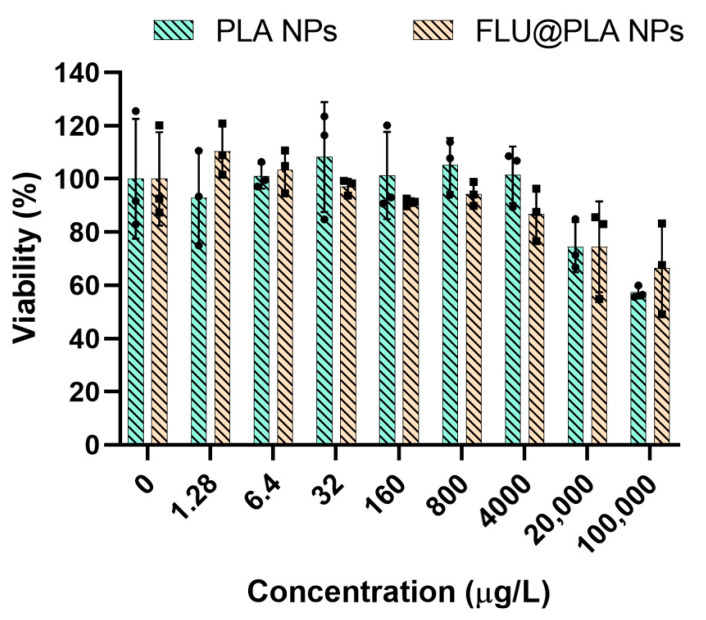
Cell viability of HCT-116 cell line incubated with different concentrations of PLA NPs and FLU@PLA NPs for 24 h. Error bars correspond to the standard deviation. Dots on the graph represent the values obtained for each experiment, n = 3.

The biocompatibility assessment of the PLA and FLU@PLA NPs relied on monitoring the changes in the mitochondrial metabolic rate of HCT-116 cells. For comparison, a control group based on pure PLA NPs was used. The MTT assay, utilizing a yellow salt, undergoes a color change to purple when reduced by NADH-dependent enzymes within mitochondria. Consequently, heightened metabolic activity results in a more intense purple coloration of the microplate wells [47,48]. The HCT-116 cell viability studies were conducted with various concentrations of PLA and FLU@PLA until reaching a maximum concentration of 100,000 μg/L. As depicted in Figure 7, exposure to different concentrations of the PLA NPs showed nearly 100% cell viability. Conversely, with FLU@PLA, it was observed that the cell viability for the HCT-116 cells remained at approximately 100% for concentrations below 32 µg/L, and for higher concentrations, the viability remained above 90%. Notably, at the maximum concentration of 4000 μg/L, the HCT-116 cell viability was significantly higher than 80%, a threshold commonly associated with cytotoxic effects [42]. An increase in the cytotoxicity for both the PLA and FLU@PLA NPs was observed for concentrations higher than 4000 μg/L.

## 3. Conclusions

In summary, our current study highlighted the potential of using the nanoprecipitation method for the controlled gelification of FLU@PLA NPs. Here, several experimental parameters were optimized, such as the concentration of PLA and PVA, the polymeric molecular weight, and the best stirring method. When comparing the PLA molecular weights, it was observed that a polymer with a lower Mw led to the distribution of NPs with improved monodispersity. This outcome was attributed to the enhanced stability and rheological properties inherent to this type of polymer. Additionally, tip sonication and ultra-turrax homogenization proved to be the most effective methods for synthesizing smaller NPs with a size distribution in the range of 200 and 500 nm. The former provided a more stable suspension, with the trade-off being a slightly larger and highly polydisperse distribution of the NPs. The PLA concentration did not significantly impact the size and morphology of the obtained NPs. However, concentrations of 10 mg/mL were required to achieve monodisperse suspensions (PLA_10_). The addition of PVA was found to be critical to hindering the gelification of large agglomerates. A concentration of 2.5 mg/mL was found optimal for the obtention of lower-PDI NPs, with higher PVA contents resulting in similar average sizes but significantly higher PDI values. However, it is important to notice that using lower PVA concentrations facilitated the purification of the PLA NPs. Nonetheless, PVA was found to reduce the ζ-potential, potentially compromising the particle stability. This concentration-dependent effect requires further investigation to ascertain an optimal threshold where the ζ-potential can be maximized without substantially compromising monodispersity. Additionally, the methodology developed for the gelification of PLA NPs demonstrated the ability to efficiently encapsulate hydrophobic drugs like FLU while ensuring that the NPs maintained sizes within the range of 200 to 500 nm. Indeed, it was found that the PLA NPs were able to accommodate a maximum of approximately 44% FLU. Considering that less than 50% FLU is effectively encapsulated in the nanocarriers, the encapsulation degree is a prominent aspect to optimize in a future study, as it would result in heightened cytostatic action without having to increase the number of delivered NPs. The viability assays showed that the FLU@PLA NPs did not exhibit cytotoxic effects up to the maximum concentration studied, 4000 μg/L. Future studies will focus on monitoring the uptake of the FLU@PLA NPs by cells and assessing their ability to release FLU in a controlled manner, potentially inducing cytotoxic effects. Nevertheless, these initial findings are promising, presenting new prospects for utilizing biodegradable polymers in drug encapsulation.

## 4. Materials and Methods

### 4.1. Chemicals, Materials, and Cells

The poly(L-lactide) (Mw = 85,000–160,000, Sigma-Aldrich, Sintra, Portugal), poly(L-lactide) (Mw = 5000, PDI ≤ 1.2, Sigma-Aldrich), poly(vinyl alcohol) (Mw~85,000–124,000, 87–89% hydrolyzed, Sigma-Aldrich), dichloromethane (99.8%, Fisher Chemical, Leicestershire, England), poly(ethylene glycol) (Mw~8000, Sigma-Aldrich), and flutamide (>98.0%, Tokyo Chemical Industry, Tokyo, Japan) were used as received. HCT-116 (ECACC: 91091005; ATCC: CCL-247), a human epithelial cell line from colon carcinoma, was used as the biological model. The cell line was kindly provided by Dr. João Carvalho of The Netherlands Cancer Institute (Amsterdam, The Netherlands).

### 4.2. Preparation of PLA NPs

The PLA NPs were synthesized using a modified nanoprecipitation method, as described by Yu et al. [49]. Initially, 10 mg of PLA was added to 1 mL of CH_2_Cl_2_ and 25 mg of PVA to 10 mL of distilled water. Both solutions were kept under magnetic stirring until the polymers were fully dissolved. Afterward, the PLA solution was added dropwise to the PVA solution using a syringe with a needle (Omnifix 30 G, 0.3 × 25 mm), keeping the solution under magnetic stirring (400 rpm). After the gelification process, the suspension was maintained under continuous stirring overnight. Then, the NP suspension was washed using three centrifugation cycles of 5 min at 12,000 rpm. After each wash, the samples were placed in an ultrasonic bath for 3 min to redisperse the NPs. Finally, all the samples were stored in deionized water and in the fridge until further use.

Different parameters were evaluated to find the optimal conditions that produced stable and monodisperse NPs. In the first assay, the effect of the stirring method was studied using conventional magnetic stirring, an ultra-turrax (12,000 rpm, 5 min), and a tip sonicator (Sonics Vibra-Cell sonifier, Newtown, CT, USA) (9W, 40% amplitude, 5 min). Then, the effects of the PVA concentration (0, 0.1, 2.5, and 10 mg/mL) and the PLA concentration (1, 10, and 50 mg/mL) were analyzed. Finally, the effect of the PLA’s molecular weight (high Mw and low Mw) was also assessed.

### 4.3. Preparation of FLU@PLA NPs

The gelification of the FLU@PLA NPs was carried out by dissolving 25 mg of FLU with 50 mg of PLA (2:1 PLA:FLU ratio) in 1 mL of CH_2_Cl_2_. The remaining procedure was followed as described above, replacing the magnetic stirring with tip sonication (tip sonicator, 9 W, 40% amplitude, 5 min). Finally, the suspension was left under continuous stirring overnight, after which 15 mg of PEG was added. For comparative purposes, pristine PLA NPs were prepared following an identical procedure but without the FLU. After washing the NP suspension, the supernatant was collected for posterior quantification of the free FLU using spectrophotometry. The FLU@PLA NP aqueous suspension was stored in the fridge at 4 °C until further use.

### 4.4. Characterization Methods

Dynamic light scattering (DLS) was used to study the hydrodynamic particle size, polydispersity index (PDI), and ζ-potential of the synthesized PLA NPs. The measurements were performed using a Malvern Zetasizer Nano-= ZS (Malvern Panalytical, Moreira, Portugal) at room temperature in samples with a medium viscosity of 0.8872 cP and a refractive index of 1.47. All the measurements were performed in triplicate. All the NPs formed were visualized using scanning electron microscopy (SEM) to analyze their morphology and size distribution. The SEM images were acquired using a field emission gun (FEG) SEM Hitachi SU-70 microscope (Hitachi High-Technologies Corporation, Tokyo, Japan) operated at 4 kV or an SEM Hitachi S4100 operated at 25 kV. For SEM observation, all the samples were prepared by placing one drop directly onto a square of carbon tape with glass positioned in an aluminum holder and allowing the solvent to evaporate. To calculate the NPs’ size, at least 100 particles were measured in each sample using the Fiji image processing software (version 2.15.1). The amount of encapsulated FLU was determined using ultraviolet-visible (UV-vis) spectroscopy, using a standard curve prepared using 5 standard FLU concentrations. Ethanol was used as the solvent since FLU is not soluble in water.

### 4.5. Cell Culture and Viability Assay

A cell culture approach was used to evaluate the biological effect of the PLA NPs and FLU@PLA NPs. The cell line used in this work was HCT-116 (ECACC: 91091005; ATCC: CCL-247), and the cells were routinely cultured in McCoy’s 5A supplemented with 2 mM of L-Glutamine, 10% Fetal bovine serum, 100 U/mL of Penicillin Gm, and 100 µg/mL of Streptomycin (complete media). The cells were maintained in an incubator at 37 °C and a 5% CO_2_ atmosphere.

To evaluate the cytotoxicity of the particles, a metabolic viability assay was performed using MTT (3-(4,5-dimethylthiazol-2-yl)-2,5-diphenyltetrazolium bromide, CAS 298-93-1, purity > 98.0%, TCI^®^ Europe, Zwijndrecht, Belgium) following the protocol from the National Institute of Health (NIH). Briefly, the HCT-116 cells were plated at a density of 1 × 10^4^ cells per well onto a 96-well clear plate and allowed to adhere overnight. These cell densities were based on preliminary assays that assessed the cell growth rate and optimal absorbance of MTT. The cells were exposed for 24 h to 8 different concentrations with a 5-fold increase in the PLA NPs and FLU@PLA NPs, starting at 1.28 µg/L and reaching up to 4000 µg/L.

The MTT (Sigma-Aldrich) was dissolved in 1× PBS (phosphate-buffered saline solution), at a pH of 7.36, to obtain a stock solution of 5 mg/mL, sterilized through filtration (0.22 μm pore PES filter), aliquoted, stored at −20 °C, and protected from light. After each exposure period, the test medium was removed, and the cells were carefully washed with 1× PBS, at a pH of 7.36. MTT was further diluted 1:10 in 1× PBS at a pH of 7.36 and added to the plate well, with an incubation period of 1 h. After the incubation period, the MTT solution was removed, and formazan crystals were solubilized through the addition of DMSO. The absorbance of the samples was measured using a microplate reader (Multiskan Spectrum—Thermo Scientific, Porto Salvo, Portugal) at a maximum absorbance of 570 nm and with 690 nm as the baseline. Viability was expressed as the percentage of solvent control [48].

## Figures and Tables

**Table 1:** Hydrodynamic diameter, PDI, and ζ-potential (with respective pH) values for the samples prepared using different stirring methods.

Sample	Diameter (nm)	PDI	Zeta Potential (mV)	pH
PLA TS	538 ± 265	0.243	−26.0 ± 1.3	6.15
PLA UT	365 ± 137	0.141	−10.1 ± 0.2	6.47

**Table 2:** Hydrodynamic diameter, PDI, and ζ-potential (with respective pH) values for the samples prepared using different PLA concentrations.

Sample	Diameter (nm)	PDI	Zeta Potential (mV)	pH
PLA_1_	287 ± 9	0.218	−18.4 ± 1.7	6.50
PLA_10_	267 ± 1	0.089	−13.4 ± 0.4	6.17
PLA_50_	295 ± 1	0.147	−12.6 ± 0,3	6.20

**Table 3:** Hydrodynamic diameter, PDI, and ζ-potential (with respective pH) values for the samples prepared using different PVA concentrations.

Sample	Diameter (nm)	PDI	Ζeta Potential (mV)	pH
PVA_0.1_	482 ± 26	0.374	−25.7 ± 0.4	6.45
PVA_2.5_	336 ± 2	0.161	−14.0 ± 0.3	6.38
PVA_10_	502 ± 6	0.209	−17.0 ± 0.2	6.27

## Data Availability

The data presented in this study are available on request from the corresponding author.

## Supplementary Materials

The following supporting information can be downloaded at: https://www.mdpi.com/article/10.3390/gels10040274/s1, Figure S1. SEM micrograph of PLA MS sample; Figure S2. The gel formed in the absence of PVA as the surfactant; Figure S3. SEM micrograph of A) PVA_0.1_ (with respective digital image), B) PVA_2.5_, and PVA_10_ samples at lower magnification (×250); Figure S4. SEM image of FLU@PLA NPs at a magnification of 60kx; Figure S5. Calibration curve of Flutamide (0.005–0.1 mg/mL) at 300 nm; Table S1—Values of FLU concentration, FLU mass, and percentages of FLU released in each wash supernatant and overall.
