# Supplementary material for: Optimized Synthesis of Poly(Lactic Acid) Nanoparticles for the Encapsulation of Flutamide
Source: Gels. 2024 Apr 18;10(4):274. doi: 10.3390/gels10040274 (PMC11049099; doi:10.3390/gels10040274)
Supplement: Supplementary file 1 [file gels-10-00274-s001.zip › gels-2954388-supplementary.pdf]

Optimized Synthesis of Poly(Lactic Acid) Nanoparticles for the Encapsulation of Flutamide

Duarte Almeida [1,2,†], Mariana Dias [3,†], Beatriz Teixeira [4], Carolina Frazão [4], Mónica Almeida [4], Gil Gonçalves [1,2], Miguel Oliveira [4,*] and Ricardo J. B. Pinto [3,*]

[1] TEMA—Centre for Mechanical Technology and Automation, Department of Mechanical Engineering,
University of Aveiro, Campus de Santiago, 3810-193 Aveiro, Portugal; duarte99@ua.pt (D.A.);
ggoncalves@ua.pt (G.G.)
[2] Intelligent Systems Associate Laboratory (LASI), 4800-058 Guimarães, Portugal
[3] CICECO—Aveiro Institute of Materials, Department of Chemistry, University of Aveiro,
Campus de Santiago, 3810-193 Aveiro, Portugal; marianamdias@ua.pt
[4] CESAM—Centre for Environmental and Marine Studies, Department of Biology, University of Aveiro,
Campus de Santiago, 3810-193 Aveiro, Portugal; beatrizteixeira99@ua.pt (B.T.);
carolina.frazao@ua.pt (C.F.); monica.alm@ua.pt (M.A.)
* Correspondence: migueloliveira@ua.pt (M.O.); r.pinto@ua.pt (R.J.B.P.)
† These authors contributed equally to this work.

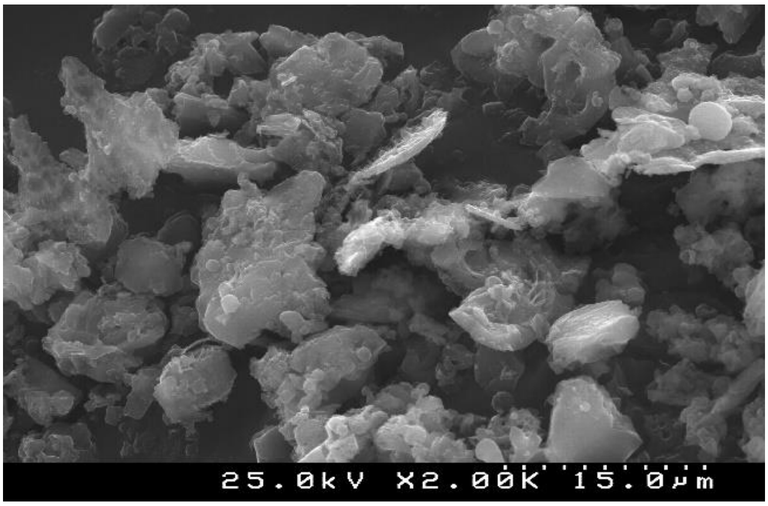

Figure S1. SEM micrograph of PLA MS sample.

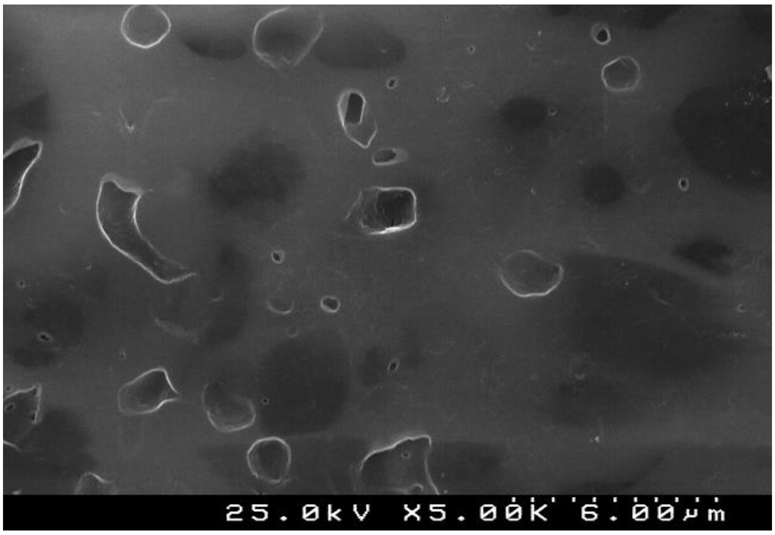

Figure S2. The gel formed in the absence of PVA as the surfactant.

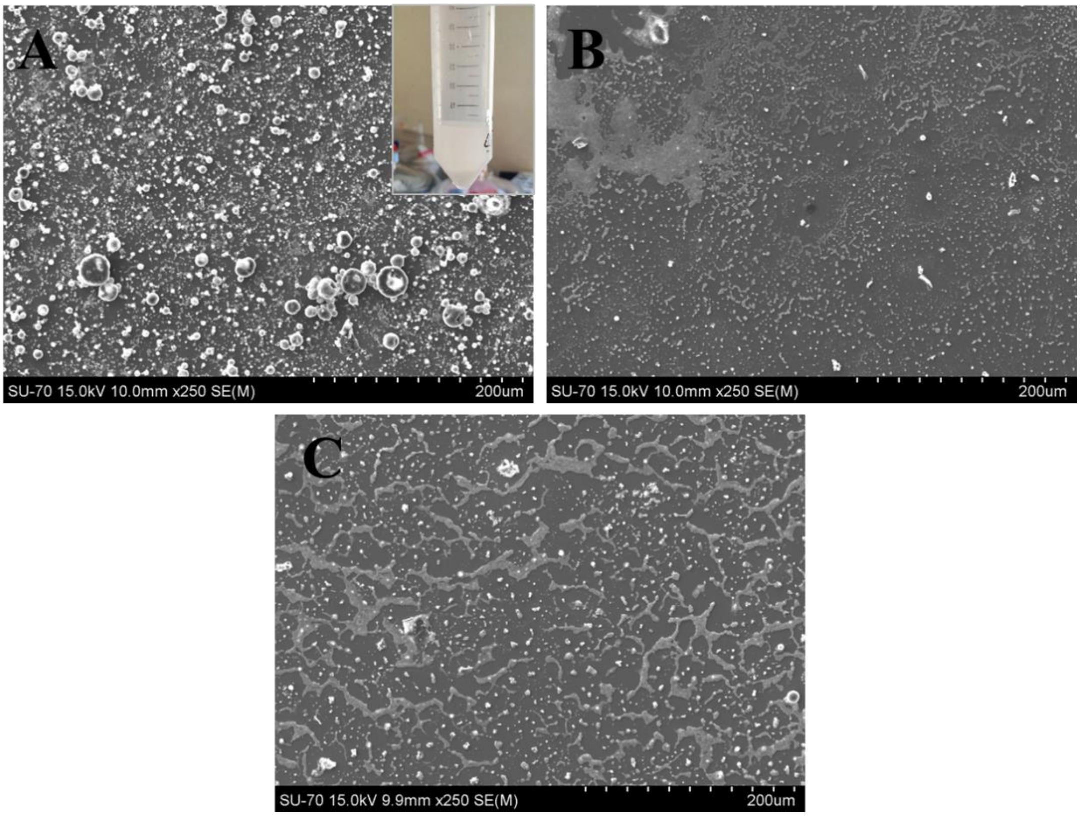

Figure S3. SEM micrograph of A) $PVA_{0.1}$ (with respective digital image), B) $PVA_{2.5}$, and PVA_{10} samples at lower magnification (250x).

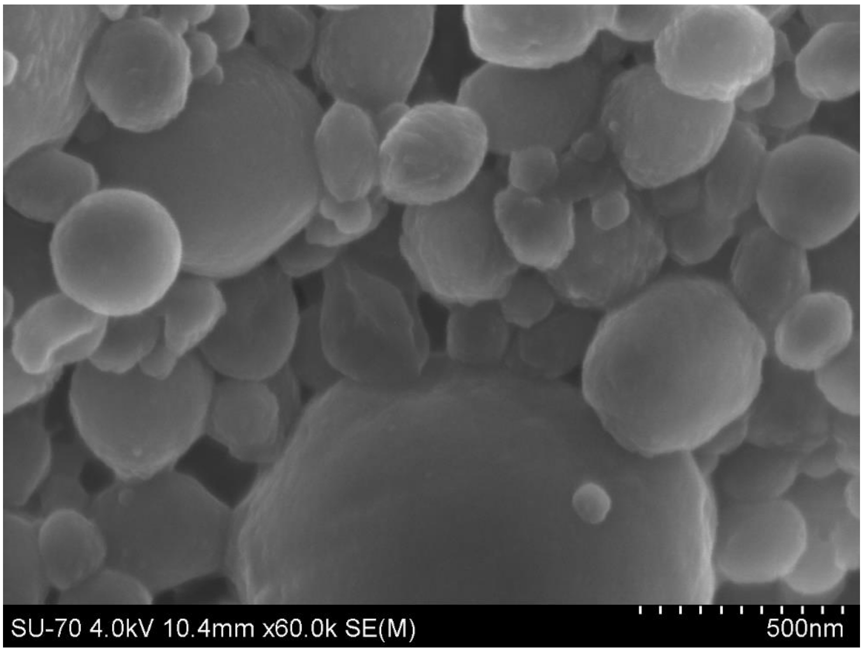

Figure S4. SEM image of FLU@PLA NPs at a magnification of 60kx.

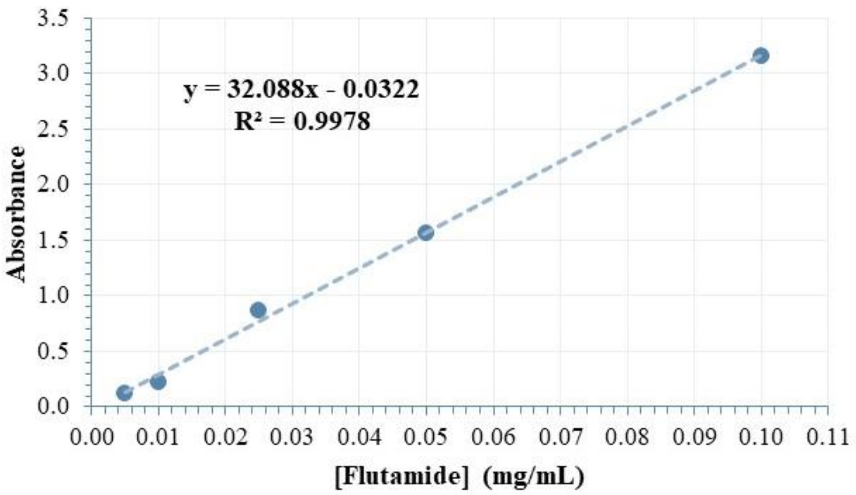

Figure S5. Calibration curve of Flutamide (0.005-0.1 mg/mL) at 300 nm.

Table S1 – Values of FLU concentration, FLU mass, and percentages of FLU released in each wash supernatant and overall.

Sample	[FLU] (mg/mL)	m_{FLU} (mg)	% FLU	% Total released
Supernatant 1	2.54	12.7	50.8	56.1
Supernatant 2	0.0423	0.211	0.846	

Supernatant 3	0.0537	0.484	1.93	
Supernatant 4	0.0689	0.620	2.48	
